# Structure-Dependent Interfacial Properties of Chaplin F from *Streptomyces coelicolor*

**DOI:** 10.3390/biom7030068

**Published:** 2017-09-19

**Authors:** Mina Dokouhaki, Emma L. Prime, Andrew Hung, Greg G. Qiao, Li Day, Sally L. Gras

**Affiliations:** 1 Department of Chemical Engineering and The Bio21 Molecular Science and Biotechnology Institute, The University of Melbourne, Parkville, VIC 3010, Australia; m.dokouhaki@student.unimelb.edu.au; 2 Institute for Frontier Materials, Deakin University, Geelong, VIC 3216, Australia; emma.prime@deakin.edu.au; 3 Polymer Science Group, Department of Chemical Engineering, The University of Melbourne, Parkville, VIC 3010, Australia; gregghq@unimelb.edu.au; 4 School of Science, RMIT University, Melbourne, VIC 3001, Australia; andrew.hung@rmit.edu.au; 5 AgResearch Ltd, Grasslands Research Centre, Tennent Drive, Palmerston North 4442, New Zealand; Li.Day@agresearch.co.nz; 6 The ARC Dairy Innovation Hub, The University of Melbourne, Parkville, VIC 3010, Australia

**Keywords:** self-assembly, pressure/area isotherms, circular dichroism, atomic force microscopy, Brewster angle microscopy

## Abstract

Chaplin F (Chp F) is a secreted surface-active peptide involved in the aerial growth of *Streptomyces*. While Chp E demonstrates a pH-responsive surface activity, the relationship between Chp F structure, function and the effect of solution pH is unknown. Chp F peptides were found to self-assemble into amyloid fibrils at acidic pH (3.0 or the isoelectric point (pI) of 4.2), with ~99% of peptides converted into insoluble fibrils. In contrast, Chp F formed short assemblies containing a mixture of random coil and β-sheet structure at a basic pH of 10.0, where only 40% of the peptides converted to fibrils. The cysteine residues in Chp F did not appear to play a role in fibril assembly. The interfacial properties of Chp F at the air/water interface were altered by the structures adopted at different pH, with Chp F molecules forming a higher surface-active film at pH 10.0 with a lower area per molecule compared to Chp F fibrils at pH 3.0. These data show that the pH responsiveness of Chp F surface activity is the reverse of that observed for Chp E, which could prove useful in potential applications where surface activity is desired over a wide range of solution pH.

## 1. Introduction

The short chaplins (Chp D–H) are small peptides ~50–60 residues in length secreted by *Streptomyces coelicolor* that feature a hydrophobic domain, containing ~40 residues [1]. The presence of both hydrophobic and hydrophilic residues facilitates adsorption at the air/water interface [2], leading to the formation of an amphipathic membrane [3] that lowers the surface tension from 72 mN/m to 26 mN/m [4,5]. The chaplins also self-assemble into functional amyloid fibrils at the surface of hyphae, increasing hydrophobicity and assisting aerial growth [6]. These properties make the chaplins potential candidates for use as emulsifiers in food products or as surface coatings in medical applications [7].

Chaplin F is expressed only during aerial growth of *S. coelicolor* and is involved in the formation of fibrils at the surface of aerial structures [6,8]. Unlike Chp E, which has no cysteine residues, Chp F and the other three short chaplins (D, G, and H) contain two cysteine residues in their primary sequence [1], which can form an intramolecular disulfide bond [1,4,5]. The importance of cysteine residues in the fibril assembly of chaplins has been demonstrated in vivo, where substitution of the cysteine residues in Chp H with glycine and valine, the residues found in Chp E that replace cysteine, compromised the assembly of fibrils on the aerial surfaces of *S. coelicolor* [9]. Disulfide bonds do not appear to be a prerequisite for fibril formation of the chaplins in vitro, however, as the reduction of the disulfide bond in synthesized Chp D, F, G, and H was found to have no effect on fibril formation by these peptides [5]. These differences between in vivo and in vitro experiments demonstrate that our understanding of cysteine residues in the assembly of chaplin fibrils is still incomplete. Chp F and Chp E also differ in their isoelectric point (pI), although they share 56.4% sequence homology [5]. A low pI of ~4 was predicted for Chp F, while Chp E has a predicted pI of 6.82 [5], confirmed experimentally as 6.7 ± 0.3 [10].

A mixture of chaplins extracted from the cell wall of *S. coelicolor* is known to lower the surface tension of water in a pH-dependent manner [7,11]. At a high pH (e.g., above 11), a mixture of chaplins exhibits a high surface activity compared to that at pH 2 or 7, leading to a greater reduction of surface activity [7]. It was proposed that differences in the surface activity of the chaplin mixture were due to a pH-induced self-assembly of these peptides into amyloid fibrils, although no experimental data has yet shown the formation of fibrils and structure of a mixture of chaplins at the various pH across which they demonstrate their surface activities [7].

Our previous study showed that altering the pH can lead to different secondary structures in Chp E, each with distinct interfacial properties [11]. At pH 3.0, Chp E was mostly monomeric or present as small oligomers with a random coil structure [10] capable of forming ordered structures at the interface that lowered the air/water surface tension to 42 ± 2 mN/m [11]. Increasing the pH to 6.7 (the pI of Chp E) or 10.0 resulted in a change in the net charge and the distribution of charges over the peptide sequence, leading to the self-assembly of Chp E into β-sheet-rich amyloid fibrils [10], which oriented randomly at the interface [11]. This structural transition was accompanied by a significant reduction in the surface activity of Chp E [11]. Differences in peptide sequence between Chp F and Chp E lead to different sensitivities to pH, including a different pI (~4 vs. ~7) and a different charge distribution across the pH range, potentially resulting in the formation of different structures. This could have a significant effect on the function of these two peptides.

In this study, we aimed to examine the self-assembly and structure of Chp F in solution as a function of pH, continuing research that aims to characterise the properties of the individual chaplin molecules. The role of cysteine residues in the self-assembly of Chp F was also probed by reducing the single disulfide bond. The interfacial properties of Chp F were investigated using a Langmuir trough, while microscopy techniques were applied to observe the morphology and thickness of interfacial Chp F films. The results of this study extend our understanding of the biological role these peptides play in the differential growth of *S. coelicolor* and also illustrate the potential complementary application of these peptides as surface-active agents in food or pharmaceutical applications.

## 2. Results and Discussion

### 2.1. pH-Responsive Self-Assembly of Chp F in Solution

**Figure 1:**
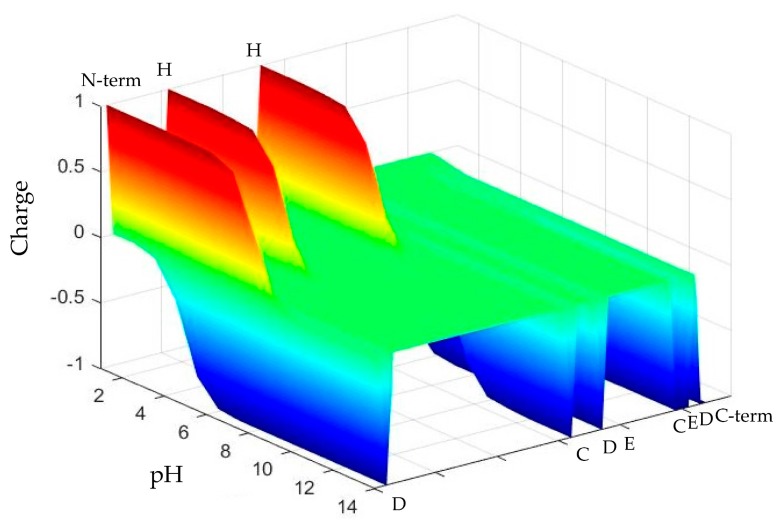
The distribution of charge (z axis) across the length of the Chaplin F (Chp F) peptide (y axis) as a function of solution pH (x axis). Chp F has 11 ionisable amino acids, including 9 residues (indicated by single letter amino acid code) and two termini (indicated by N-term and C-term). The three conditions selected result in a significantly different charge profile across the peptide.

The net charge, number of charged residues and charge distribution over the length of the Chp F peptide determined by the Henderson–Hasselbalch equation differed significantly at the three pH values chosen for this study (3.0, 4.2, and 10.0), as shown in Figure 1. These conditions correspond to the pI of Chp F, as determined here by isoelectric focusing (IEF) (4.2 ± 0.1), pH 3.0, and pH 10.0, where the peptide has an overall net charge of 0, +3, and −8, respectively. The pI determined here for Chp F is within the range of 3.80–4.49 previously predicted for this peptide using the ProtParam tool (Expert Protein Analysis System (ExPasy) server) [5,12].

**Figure 2:**
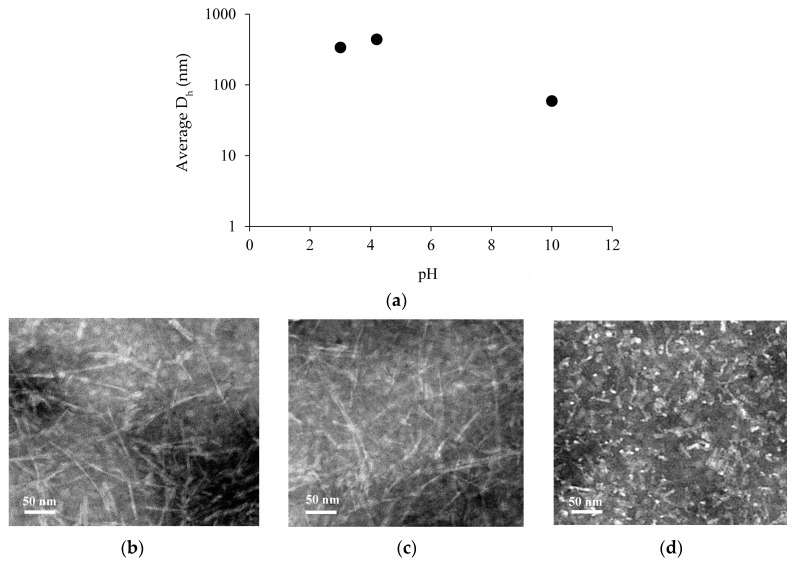
The size and morphology of Chp F assemblies as a function of solution pH: (**a**) the hydrodynamic diameter (D_h_) of Chp F at pH 3.0, the isoelectric point (pI) of 4.2, and pH 10.0. The size of the error bars, based on standard deviation, is smaller than the data series. Transmission Electron microscopy (TEM) images of Chp F samples at pH 3.0 (**b**), the pI of 4.2 (**c**) and pH 10.0 (**d**).

The hydrodynamic diameter (D_h_) of Chp F under these three pH environments was measured by dynamic light scattering (DLS), and the structural morphology of Chp F assemblies was characterised by Transmission Electron Microscopy (TEM), as shown in Figure 2. At pH 10.0, Chp F assembled into aggregates with an average D_h_ of 59 ± 6 nm (Figure 2a). In contrast, at pH 3.0 or 4.2, Chp F formed assemblies with an average D_h_ of 337 ± 20 or 439 ± 7 nm, respectively (Figure 2a), around 6–7 times larger than the structures formed under basic conditions. At the pI, Chp F formed assemblies with the highest D_h_. The lack of overall charge on the peptide at the pI likely allows the formation of larger assemblies than those formed at other pH conditions [13], where charges are present. Despite the difference in average D_h_ at pH 3.0 and 4.2, TEM revealed no significant difference in the size or morphology of these structures (Figure 2b,c). Under both conditions, intertwined assemblies composed of large fibrillar structures with a length of 60–470 nm and a width of 4–6 nm were observed (Figure 2b,c). In contrast, short assemblies with an average length of 55 nm and width of 4–6 nm were observed at pH 10.0 (Figure 2d); consistent with the smaller D_h_ measured at this pH, these samples were also more polymorphic in nature.

The fibrillar structures observed for Chp F under acidic conditions resemble those formed by a mixture of short chaplins under quiescent conditions [4,14] but not those formed when the Chp F peptide was previously vortexed [5], as vortexing may have affected assembly both by assisting protein diffusion and increasing protein denaturation at the air/water interface [15,16,17]. The mixed peptides formed fibrils typically spanning several hundred nanometers in length with a width of 4–6 nm, similar to the Chp F fibrillar structures observed here [4].

### 2.2. Fibril Formation of Chp F at Different pH

Two experiments were conducted to probe the differences in Chp F structure as a function of pH: circular dichroism (CD) was used to examine secondary conformation and a Thioflavin-T (ThT) binding assay was conducted as a measure of fibril formation.

**Figure 3:**
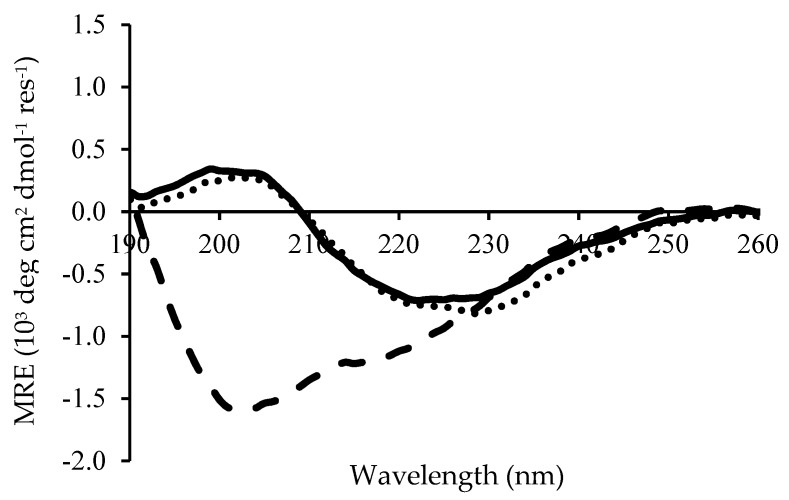
Secondary structure of Chp F assessed by circular dichroism (CD) for samples freshly prepared at pH of 3.0 (solid line), the pI of 4.2 (dotted line), or pH 10.0 (dashed line). The data is the average of three scans. MRE: Mean Residue Ellipticity.

Circular Dichroism demonstrated that the secondary structure of Chp F changes as a function of solution pH, as shown in Figure 3. Similar CD spectra were obtained for Chp F at pH 3.0 or 4.2 (pI), with a small positive peak at 202 nm and a large negative peak at 225 nm. This is indicative of β-sheet conformation, which typically gives a positive peak near 205 nm and a negative peak in the range 210–230 nm [18,19,20]. In contrast, at pH 10.0, the spectrum for Chp F showed two negative peaks, one at ~202 nm and the other at ~220 nm, with no positive peak. This suggests a mixture of β-sheet and random coil conformation at basic pH. The shift of the CD spectrum toward a large negative peak at ~202 nm is a typical feature of a random coil structure [20,21]. The program Dichroweb was used to deconvolute the secondary structure obtained by CD and the results are summarised in Table 1. This data confirms that Chp F has a considerable amount of β-sheet structure at pH 3.0 (60%) or at pH 4.2 (64%). The amount of β-sheet was approximately halved at pH 10.0 (30%), with an increase in random coil structure observed.

**Figure 4:**
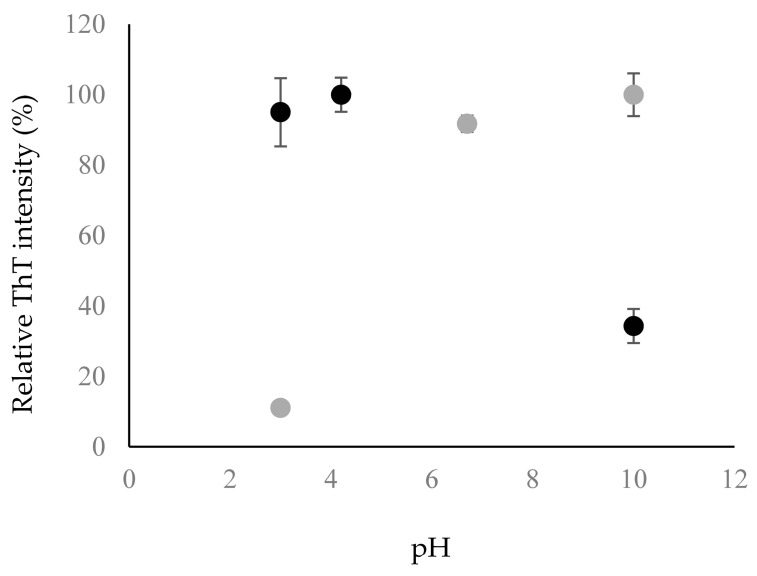
Fluorescence emission of Thioflavin-T (ThT) at a wavelength of 482 nm in Chp F (black) and Chp E (grey) solutions at different pH conditions; pH 3.0, pI of 4.2 for Chp F and 6.7 for Chp E and pH 10.0. The data presented is the average of two trials performed on two separate occasions and error bars are the standard deviations.

This observation was further corroborated when the fluorescence intensity of ThT was measured to be higher at acidic pH compared to basic pH (Figure 4). ThT is known to bind to the β-sheet structure of amyloid fibrils, leading to a rise in fluorescence intensity when more β-sheet structure is present [22]. The ThT intensity of Chp F was similar at pH 3.0 and 4.2 but it was ~3-fold lower at pH 10.0 (Figure 4). This indicates a lower amount of β-sheet-containing amyloid fibrils at pH 10.0, consistent with the CD data (Figure 3, Table 1).

**Figure 5:**
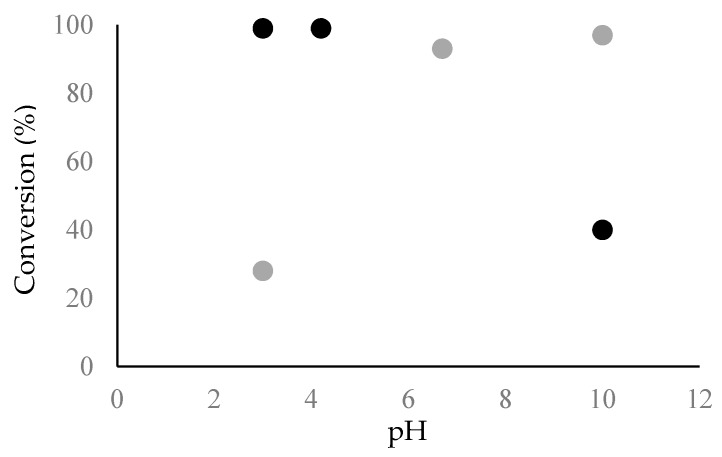
The conversion of Chp F (black) or Chp E (grey) peptides into fibrils as a function of solution pH (pH 3.0, pI of 4.2 for Chp F and 6.7 for Chp E and pH 10.0) measured by amino acid analysis. The data for Chp E is reproduced from Dokouhaki et al. [10].

The higher fibril formation at acidic pH compared to basic pH was further confirmed quantitatively by amino acid analysis, as shown in Figure 5. At pH 3.0 and 4.2, a higher level of the Chp F peptide was converted into insoluble material (~99%) compared to pH 10.0 (~40%). These data also confirm that fibril assembly is rapid for Chp F and occurs within the dead time of the experiment (~10 min), especially under acidic conditions. Rapid self-assembly into fibrils has also been found for other individual short synthetic chaplins [5,10] and a mixture of chaplins extracted from the cell wall of *S. coelicolor* [5].

The CD and ThT data suggest that the differences in net charge, the number of charged residues and the distribution of these charges over the peptide at different pH lead to assemblies with different structures. The C-terminal end of the Chp F peptide theoretically has a negative charge at pH 10.0, as shown in Figure 1. The negative charge on this terminus and seven other residues could lead to electrostatic repulsion between peptides, reducing the alignment of Chp F and reducing the association of peptides that leads to fibril formation. In contrast, the decrease in the theoretical number of negatively charged residues and changes in the distribution of charges over the whole length of the peptide under acidic conditions (Figure 1) could reduce the electrostatic repulsion between Chp F peptides. This could allow better alignment along the peptide chain and promote the formation of fibrils rich in β-sheet conformation.

A comparison with our previously reported results for another short chaplin, Chp E [10], further corroborates this observation. At pH 3.0, the N-terminal end of Chp E is positively charged (+6) and a random coil structure was observed [10]. At conditions of higher pH (6.7 or 10.0), the overall net charge and the number of positively charged residues along the peptide is reduced and fibrils rich in β-sheet structure were observed [10].

Interestingly, the solution pH has the opposite effect on the structure and fibril assembly for Chp E and Chp F. As can be seen from the ThT fluorescence assay and amino acid analysis data in Figure 4 and Figure 5, in the case of Chp E, the transition from assemblies with a random coil structure to fibrils with a β-sheet structure is induced by an increase in pH, while for Chp F this transition is achieved by a reduction in pH. This is likely due to differences between the sequences of these two peptides and their complementary charge distribution, which consequently affects the self-assembly process.

### 2.3. Cysteine-Independent Fibril Formation of Chp F

Chaplin F contains two cysteine residues that are also present in Chp D, H, and G but absent in Chp E [1]. These residues are thought to form an intramolecular disulfide bond [1]. To assess the role of this disulfide bond in fibril formation, we probed the ability of reduced Chp F to adopt β-sheet conformation at pH 3.0, 4.2 (pI), and 10.0. The peptide was reduced with tris(2-carboxyethyl)phosphine (TCEP) and mass spectrometry was used to confirm the reduction and corresponding 2 Da increase in molecular weight (Figure S1). There was no difference between the secondary structure determined by CD for reduced and non-reduced Chp F (Figure S2), suggesting that disulfide bond formation is not necessary for assembly into the β-sheet structure required for Chp F fibrils in vitro. This is in agreement with previous experiments, which found that a disulfide bond is not a prerequisite for β-sheet formation for Chp F and the other cysteine containing sequences, Chp D, G, and H, at pH 7.0 [5]. The finding is in contrast, however, to in vivo experiments [9], suggesting that additional complexity is involved in the secretion, interaction or assembly of these peptides in vivo.

### 2.4. Interfacial Properties of Chp F as a Function of pH

**Figure 6:**
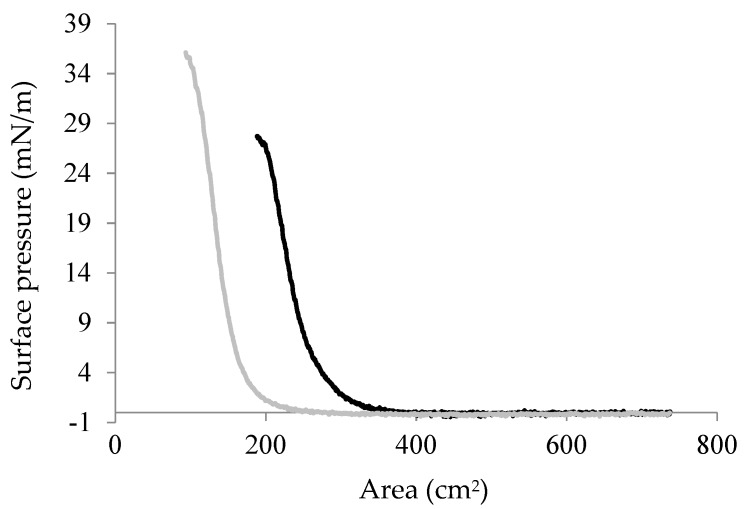
Surface pressure/area (π/A) isotherms for Chp F films formed at pH 3.0 (black line) or at pH 10.0 (grey line). The data is representative of two experiments.

The influence of pH on the interfacial assembly and possible function of Chp F as a surface-active agent was investigated by performing pressure/area (π/A) isotherms on a Langmuir trough. The isotherms for Chp F films at the air/water interface at pH 3.0 and 10.0 are shown in Figure 6, as large differences were observed between the secondary structure, fibril assembly and fibril morphology of Chp F samples under these conditions. Both isotherms contain three different states. Upon compression, the surface pressure increased gradually from a gas state, where molecules are far apart, to a liquid state, where molecules are in close proximity with little spatial freedom and finally to a solid state, where the interfacial film is fully compressed [23]. Molecules in the solid state reached a bending point where the film collapsed if they were further compressed [23]. The bending point of Chp F was 23 ± 3 mN/m and 36 ± 2 mN/m at pH 3.0 and 10.0, respectively.

A lower surface activity was obtained at pH 3.0 compared to at pH 10.0 for the same concentration of peptide at the interface (2.17 mg/m^2^). Chp F can lower the surface tension of water from 72.8 mN/m to 50 ± 3 mN/m at pH 3.0 or to 37 ± 2 mN/m at pH 10.0. There is a small but significant difference between the surface areas occupied by one Chp F molecule at each pH: 1.4 × 10^−14^ cm^2^ at pH 3.0 compared to 0.9 × 10^−14^ cm^2^ at pH 10.0, calculated as described previously using the pressure/area isotherms [24]. These data show that the surface area occupied by one Chp F molecule at pH 3.0 is approximately 1.6 times larger than that at pH 10.0, indicating a denser packing of Chp F molecules at basic pH.

The ability of proteins to lower the surface tension is known to be associated with the exposure of hydrophobic residues to the surface, facilitating adsorption to the interface [2]. At pH 10.0, Chp F showed higher surface activity compared to at pH 3.0, likely due to the increased interfacial exposure of the hydrophobic areas of the protein due to the higher proportion of unaggregated material and reduced level of β-sheet conformation at this pH. Sawyer et al. [5] have previously reported higher surface activity for Chp F when the peptide was less aggregated, where the pendant droplet method was used to measure the change in surface tension from 72.8 mN/m to 28 ± 3 mN/m induced by Chp F at pH 6.8 [5]. This change in surface tension is similar to that observed here.

The self-assembly of Chp F into fibrils at pH 3.0, which probably occurs through hydrophobic residues, likely decreases the hydrophobic area in contact with the interface, reducing the surface activity of the peptide under acidic conditions. This observation is again consistent with the previous work of Sawyer et al., who observed a decrease in surface activity when Chp F fibrils were formed by vortexing or induced by high concentrations of peptide [5]. The surface activity observed here for Chp F as a function of pH is also consistent with the pH-dependent surface activity observed previously for a mixture of short chaplins [7]. In this case, an increase in solution pH to above 11 resulted in higher surface activity for the mixture of short chaplins, compared to pH 7 or 2 [7]. Since there is no difference in the net charge, number of charges, or the distribution of charges over the length of Chp F at pH ≥10, it can be assumed that the structure of Chp F remains as a mixture of random coil and β-sheet at pH ≥10, resulting in higher surface activity at this range of pH (10–14) compared to a lower pH of 3.0 or 4.2.

### 2.5. Morphology of Chp F Interfacial Films

**Figure 7:**
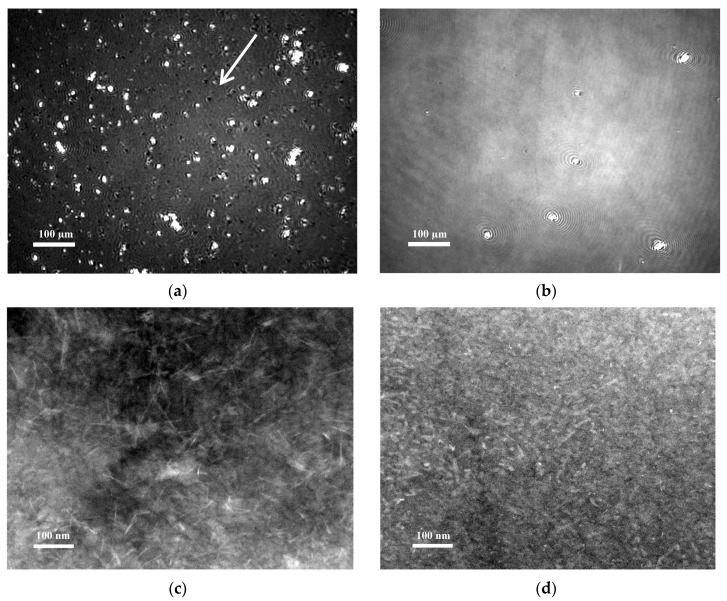
The morphology of Chp F films formed at the air/water interface observed by Brewster angle microscopy (BAM) (**a**,**b**) and TEM (**c**,**d**) at pH 3.0 (**a**,**c**) or pH 10.0 (**b**,**d**). The arrows show the micro pores that can be observed in selected films in the fibrous structures.

The relationship between pH-induced structural changes and the surface activity of Chp F was further explored using Brewster angle microscopy (BAM) to observe the morphology of Chp F films in situ, as the films were compressed to their bending points. Distinct differences were observed between the Chp F interfacial films formed at pH 10.0 and 3.0, as shown in Figure 7a,b. A homogenous film was observed for Chp F at pH 10.0 (Figure 7b), while the film formed at pH 3.0 (Figure 7a) contained numerous small dense aggregates distributed over the film together with micropores (shown with the arrow in Figure 7a). Significantly fewer aggregates were seen at pH 10.0, consistent with the lower propensity of Chp F peptides to aggregate at high pH compared to low pH.

In order to further investigate the morphology of the Chp F films, these films were transferred to the surface of carbon-coated grids using the horizontal Langmuir–Schaefer dipping method. TEM images of these films (Figure 7c,d) showed that Chp F is present as long, thin fibrils at the interface at pH 3.0, similar in length and width to those formed in solution at the same pH (Figure 2b). The structures formed at pH 10.0 are shorter than those formed in solution (less than 60 nm) but of similar width (4–6 nm) (Figure 7d). At both pH conditions, the structures formed are randomly arranged at the interface. The more homogenous film formed at pH 10.0, as shown in both BAM and TEM, may reflect a better packing of the short structures observed at pH 10.0 with a smaller area occupied at the interface (Figure 6) compared to the large fibrils present at pH 3.0.

In order to compare film thickness and roughness under different pH conditions, Chp F films were transferred onto a solid surface, dehydrated and then analysed by Atomic Force Microscopy (AFM). The interfacial films formed by Chp F at pH 3.0 and 10.0 were found to have a similar thickness of 1.6 ± 0.4 nm, despite the differences in morphology and roughness (Figure S3). This thickness is consistent with previous reports of a 1–3 nm thick film formed by a mixture of short chaplins on a solid surface at pH 7 [7]. The film formed at pH 3.0 containing long fibrils is ~5-fold rougher (676.15 pm vs. 136.41 pm) than the film formed at pH 10.0.

### 2.6. Comparison of Chp F to Chp E at the Air/Water Interface

A comparison of films formed by two chaplins, Chp F and Chp E, at the air/water interface, highlights numerous differences. In contrast to Chp F, a higher surface activity was found for Chp E at pH 3.0 compared to pH 10.0. Interfacial assembly of Chp E at pH 3.0 resulted in ordered structures with random coil conformation, aligned in one direction [11], while no alignment was observed for Chp F under either pH condition. At pH 10.0, the film formed from Chp E consisted of fibrils with no evidence of alignment [11], similar to the fibrils formed by Chp F at pH 3.0.

Surface-pressure isotherms showed that a smaller interfacial area was occupied by one Chp E molecule at pH 10.0 [11] compared to a Chp F molecule at pH 3.0 (0.31 × 10^−14^ cm^2^ vs. 1.4 × 10^−14^ cm^2^), where both peptides have high conversion into fibrils (97% and 99%, respectively) (Figure 5). This likely reflects a more compact self-assembly of Chp E fibrils at pH 10.0, reducing the extent of exposure of hydrophobic residues [11]. This is presumably the reason why a ~4-fold higher concentration of Chp E fibrils was required at pH 10.0 [11] compared to Chp F fibrils at pH 3.0, in order to reach the same level of surface activity. Although further work is required to determine the structure of the short chaplins, the presence of two cysteine residues in Chp F might impact the structure and thereby influence the availability of hydrophobic residues. These residues have previously been shown to be important in the structures formed by a hydrophobin molecule via the formation of a hydrophobic patch at the surface [25,26].

## 3. Materials and Methods

Immobiline dry strip gels with a linear pH gradient of 3–10 for isoelectric focusing were purchased from GE Healthcare Life Sciences (Rydalmere, NSW, Australia). Glycerol, thiourea, dichlorodiphenyltrichloroethane (DDT), and ampholyte at the corresponding pH range of 3–10 required to prepare the OFFGEL stock solution, were obtained from Agilent Technologies (Mulgrave, Victoria, Australia). ThT, acetonitrile (ACN), chloroform, TCEP, sulfuric acid (99.8% pure), and hydrogen peroxide (30% pure) were of analytical grade, obtained from Sigma-Aldrich (Castle Hill, NSW, Australia). Silicon wafers were obtained from MMRC Pty. Ltd. (Melbourne, Australia). The Chp F peptide with the sequence of DSGAQAAAAHSPGVLSGNVVQVPVHIPVNVCGNTIDVIGLLNPAFGNECEND5 (>95% purity) was synthesised by CS Bio Co (Menlo Park, CA, USA).

### 3.1. Isoelectric Focusing Gel

The 3100 OFFGEL Fractionator with a 24-well setup (Agilent Technologies) was used to determine the pI of synthetic Chp F according to the protocol provided by the supplier. Briefly, the 24 cm immobiline dry strip gels were rehydrated with rehydration solution (40 μL per well) for 10–15 min prior to sample loading, as described previously [27]. Chp F (0.1 mg) was dissolved in 3.6 mL of protein OFFGEL stock solution and 150 μL added to each of the 24 wells. The current and voltage were applied as described previously [27].

### 3.2. Preparation of Solutions

Solutions of Chp F peptide were freshly prepared at a final concentration of 0.1 mg/mL in a solvent consisting of 90% (*v*/*v*) Milli-Q water (ultra-pure water with a resistivity of 18.2 MΩ/cm at 25 °C) and 10% (*v*/*v*) ACN without vortexing or vigorous agitation. The pH of Chp F solutions was adjusted to 3.0, the pI of 4.2 (as measured with IEF), or 10.0 immediately after dissolving the peptide using 0.1 M HCl or 0.1 M NaOH.

### 3.3. Size Measurement of Chp F as a Function of pH

A 1 mL aliquot of Chp F solution was placed in a disposable capillary cell (DTS1061, Malvern Instruments Ltd., Malvern, UK). The size of the peptide in solution was measured at a fixed scattering angle of 173° at 25 °C using a Nano series zeta-sizer (Malvern Instruments Ltd.). Three replicate measurements were performed for each pH value, and the data presented is the average of these replicates with the standard deviation.

### 3.4. Circular Dichroism Spectroscopy Measurements

The secondary structure of Chp F, in solutions of different pH, was measured using CD. For the experiments performed under reducing conditions, Chp F was dissolved at a concentration of 0.1 mg/mL in a solvent consisting of 90% (*v*/*v*) Milli-Q water and 10% (*v*/*v*) ACN containing 15 mM TCEP at pH 7.0 [28,29]. The reduction was confirmed using mass spectrometry (LC/MS, Agilent esiTOFprotein, Agilent Technologies). After reduction, the pH of the Chp F solutions was adjusted to 3.0, 4.2 (pI), or 10.0 using 0.1 M HCl or 0.1 M NaOH. A 1 mm path length quartz cuvette was used at 25 °C and spectra recorded from 260 nm to 190 nm in 0.5 nm steps with a scan time of 4 s per step. A total of three scans were performed per sample.

CD data analysis was performed using CD tool software [30]. The three spectra were averaged and corrected for minor solvent contributions by subtracting the reference spectrum of pH-adjusted water containing 10% ACN and 15 mM TCEP. The data was then converted to units of mean residue ellipticity (θ) using the molecular weight of Chp F (5180 Da). DICHROWEB, a web-based calculation server, was used to determine the percentage of each secondary structure present [31]. DICHROWEB includes a wide range of protein spectral databases and various programs [31]. The CDSSTR reference set 1 (optimized for 178–260 for protein in solution) was applied in this study [32].

### 3.5. Transmission Electron Microscopy

An aliquot of 6 μL of the Chp F solution was added to a 300 mesh carbon-coated copper TEM grid (ProSciTech, Thuringowa, QLD, Australia). The grids were negatively stained with phosphotungstic acid (2% (*w*/*v*)) to increase contrast, as described previously [5]. A Tecnai G2 TF30 microscope (FEI Company, Eindhoven, The Netherlands) operating at 200 kV equipped with a 2 k × 2 k Charge Coupled Device (CCD) camera (Gatan, Pleasanton, CA, USA) was used to observe the morphology of Chp F samples immediately after they were air dried. The software Autodesk [33] was used to obtain the width and length measurements of fibrils for each pH treatment (*n* = 50).

### 3.6. Thioflavin-T Binding

The Chp F solutions were mixed with a ThT solution to reach a final concentration of 10 μM of ThT [32]. The pH of the solutions was adjusted to 3.0, the pI of 4.2, or 10.0 using 0.1 M HCl or 0.1 M NaOH, respectively. A spectrofluorometer (Varioskan Flash micro plate reader, Thermo Fisher Scientific Inc., Waltham, MA, USA) was used to perform the ThT fluorescence measurements. A slit-width of 5 nm was used for excitation and emission. Samples (200 μL) were loaded into a 96-well micro plate (OptiPlate-96, F micro plate, Perkinelmer, San Jose, CA, USA). After sample preparation, the fluorescence intensity of ThT was immediately measured at different emission (470–600 nm) and excitation (450 nm), as described previously [34]. Three replicate measurements were performed for each solution pH, and the assay repeated on two separate occasions. Variability between the three replicate measurements was low and the data presented is the average for the two sets of experiments. The ThT fluorescence intensity was also measured for Chp E solutions at pH 3.0, 10.0, or at the pI of 6.7 using the same method.

### 3.7. Amyloid Fibril Assembly

To investigate the conversion of Chp F peptides into fibrils, solutions of Chp F were freshly prepared and centrifuged immediately at 313,000× *g* for 50 min at 4 °C with a Beckman XL-centrifuge (Beckman Coulter Inc., Brea, CA, USA), as described previously [35,36]. The concentration of amino acids in the supernatant was determined with a ninhydrin-based detection technique [36].

### 3.8. Apparatus

A Teflon Langmuir trough (76 cm × 10 cm, Nima Technology Ltd., Coventry, United Kingdom) model 711D with a single Delrin barrier (11.2 cm × 1.6 cm) was used to characterize the properties of the interfacial films formed by the Chp F preparations at the air/water interface. Prior to each experiment, the trough and barrier were cleaned with chloroform and the Wilhelmy plate moistened with Milli-Q water and attached to the pressure sensor. The trough was then filled with Milli-Q water and allowed to equilibrate with the laboratory air at a temperature of 25 ± 1 °C. The water surface in the trough was then swept clean by vacuum aspiration until a zero reading was achieved on the balance, and the Chp F solution was then added to the surface.

### 3.9. Surface Pressure/Area Isotherm

Surface pressure/area isotherms were performed using the Langmuir trough prepared as described above. A Hamilton syringe was used to inject [37] the appropriate amount of Chp F solution (2.17 mg/m^2^) at pH 3.0 or 10.0 onto the surface of water at the same pH. The sample was allowed to equilibrate for 30 min before performing the surface pressure/area isotherms. The Delrin barrier was used to compress the surface film at a rate of 50 cm^2^/min while measuring the surface pressure as a function of the area (cm^2^) until the film reached the bending point [23]. The surface tension at this pressure was obtained using Equation (1) [38].

*π* = *γ_0_* − *γ* (1)

where *π* is surface pressure, and *γ_0_* and *γ* are surface tension of water and subphase including amphiphiles, respectively.

The area per molecule was calculated as previously described using established methods [23,39]. The experiment was repeated two times to ensure reproducibility of the data, and the data presented is representative of two experiments.

### 3.10. Brewster Angle Microscopy

The morphology and homogeneity of Chp F Langmuir films at pH 3.0 or 10.0 at the air/water interface was visualized using a Brewster angle microscope (BAM, KSV NIMA, Biolin Scientific, Västra Frölunda, Sweden) mounted on a Langmuir trough (KN 2003, KSV NIMA). The BAM was equipped with a 50 mW laser emitting p-polarized light with a wavelength of 658 nm, which was reflected off the air/water interface at the Brewster angle (~53°) [40]. The lateral resolution of the microscope was 2 μm. Images were obtained when the films were compressed to the pressure of the bending point.

### 3.11. Transmission Electron Microscopy of Langmuir Films

TEM was used to observe the morphology of Chp F Langmuir films under the same experimental conditions as used for BAM above. Chp F Langmuir films were horizontally deposited using the Langmuir–Schaefer method [41,42] onto 300 mesh carbon-coated copper TEM grids (ProSciTech) [3]. The excess sample was removed by blotting the edges of the grid with filter paper, and the samples were allowed to dry [3]. The sample staining, imaging, and size measurements were performed as described above.

### 3.12. Atomic Force Microscopy

The thickness and roughness of Chp F films formed at the interface was measured using AFM. Prior to the experiment, silicon wafers, which act as a hydrophilic substrate [43,44], were cut into ~1 × 1 cm pieces and cleaned with piranha solution containing sulfuric acid and hydrogen peroxide (7:3) before rinsing with Milli-Q water [45].

Chp F films were prepared on the Langmuir Trough as previously described, and compressed at a rate of 50 cm^2^/min until they reached the bending point. The film was then transferred onto the silicon substrate using the vertical Langmuir–Blodgett method, as described previously [46]. The silicon was dipped vertically at a rate of 8 mm/min through the liquid interface, and removed again at the same rate [46]. The sample deposited on the substrate was allowed to air dry.

An Asylum Research MFP-3D atomic force microscope in tapping mode and ultrasharp SiN gold-coated cantilevers (MikroMasch, Sofia, Bulgaria) were used to measure the thickness and roughness of the air-dried films on silicon wafers. The air-dried films were scanned by AFM within 24 h of transfer. Film thickness was estimated by scratch analysis (mechanical removal of the film) and by tracing a profile along the film as described previously [45]. The thickness values represent the mean of at least three measurement areas along the scratch profile with the standard deviation of the three replicate experiments (*n* = 3 ± standard deviation). Image processing and surface roughness analyses were performed using the Nanoscope (Bruker, Coventry, United Kingdom) and Igor Pro (Wavemetrics, Lake Oswego, OR, USA) software programs, respectively.

## 4. Conclusions

The self-assembly of Chp F in solution is a rapid, pH-dependent process that occurs independently of disulfide bond formation. At pH of 3.0 or the pI of 4.2, ~99% of Chp F peptide is converted into long amyloid fibrils rich in β-sheet structure. A lower conversion (~40%) to shorter fibrils was observed at pH 10.0, where a mixture of β-sheet and random coil structure was observed. Under these conditions, electrostatic repulsion between Chp F peptides from the high net negative charge, particularly around the C-terminal end, could inhibit assembly. Films formed at the air/water interface differed in activity and morphology as a function of pH: at pH 10.0, the film was homogenous, with higher surface activity and a lower area per molecule than at pH 3.0, where long fibrils and aggregates were randomly orientated in a film also containing micropores. The lower level of β-sheet conformation at pH 10.0 is likely responsible for the higher surface activity, due to increased exposure of hydrophobic Chp F residues to the interface. The pH-dependence of Chp F surface activity observed here is complementary to the behaviour of Chp E but similar to the behaviour described for a mixture of short chaplins. Insights gained here will help to identify potential applications for Chp F as a surface-active agent in food or pharmaceutical applications, and further elucidate the role of Chp F and the other short chaplins in the differential development of *S. coelicolor*.

## Figures and Tables

**Table 1:** The secondary structure of Chp F in solution at different pH determined by Dichroweb deconvolution of CD spectra.

Solution pH	β-Sheet	Random Coil
3.0	60%	6%
4.2	64%	4%
10.0	30%	36%

## Supplementary Materials

The following are available online at www.mdpi.com/2218-273X/7/3/68/s1, Figure S1: The mass spectra of Chaplin F, Figure S2: Circular Dichroism spectra of reduced Chp F, Figure S3: Atomic Force Microscopy height and deflection images of the interfacial film formed by Chp F.
