# Supplementary material for: Structure-Dependent Interfacial Properties of Chaplin F from Streptomyces coelicolor
Source: Biomolecules. 2017 Sep 19;7(3):68. doi: 10.3390/biom7030068 (PMC5618249; doi:10.3390/biom7030068)
Supplement: Supplementary file 1 [file biomolecules-07-00068-s001.pdf]

Supplementary Materials

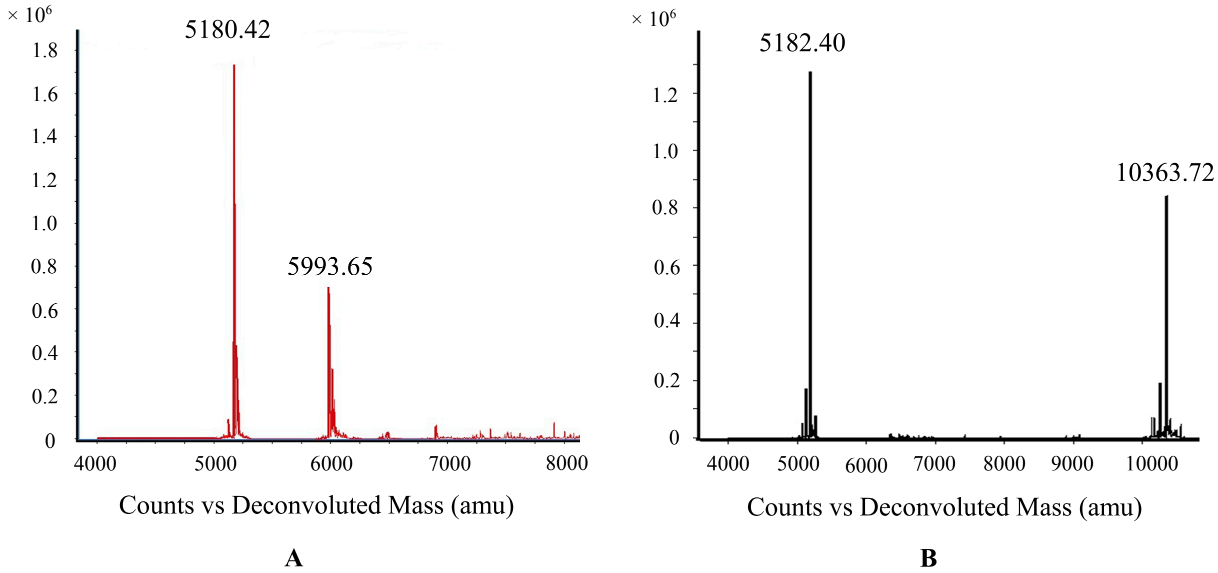

Figure S1. The mass spectra of Chaplin F (Chp F) detected by mass spectrometry. (**A**) The deconvolution of mass spectrum of Chp F where the disulfide bond is present shows a molecular weight of 5180.42 Da before reduction with tris(2-carboxyethyl)phosphine (TCEP), the second peak with the molecular weight of 5993.65 Da is thought to relate to an impurity from peptide synthesis. (**B**) The deconvolution of the mass spectrum of Chp F after reduction with TCEP revealed a 2 Da increase in molecular weight, the second peak with the molecular weight of 10363.72 Da is though to be a dimer of Chp F, which has a mass of ~5180 Da.

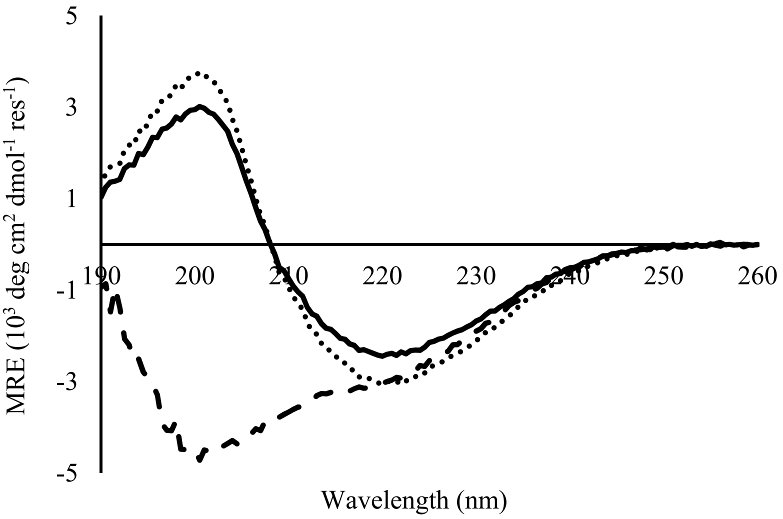

Figure S2. Circular Dichroism (CD) spectra of reduced Chp F: at a pH of 3.0 (solid line), the pI of 4.2 (dotted line) and pH 10.0 (dashed line). The data is the average of three scans. These spectra are consistent with the spectra obtained for non-reduced Chp F.

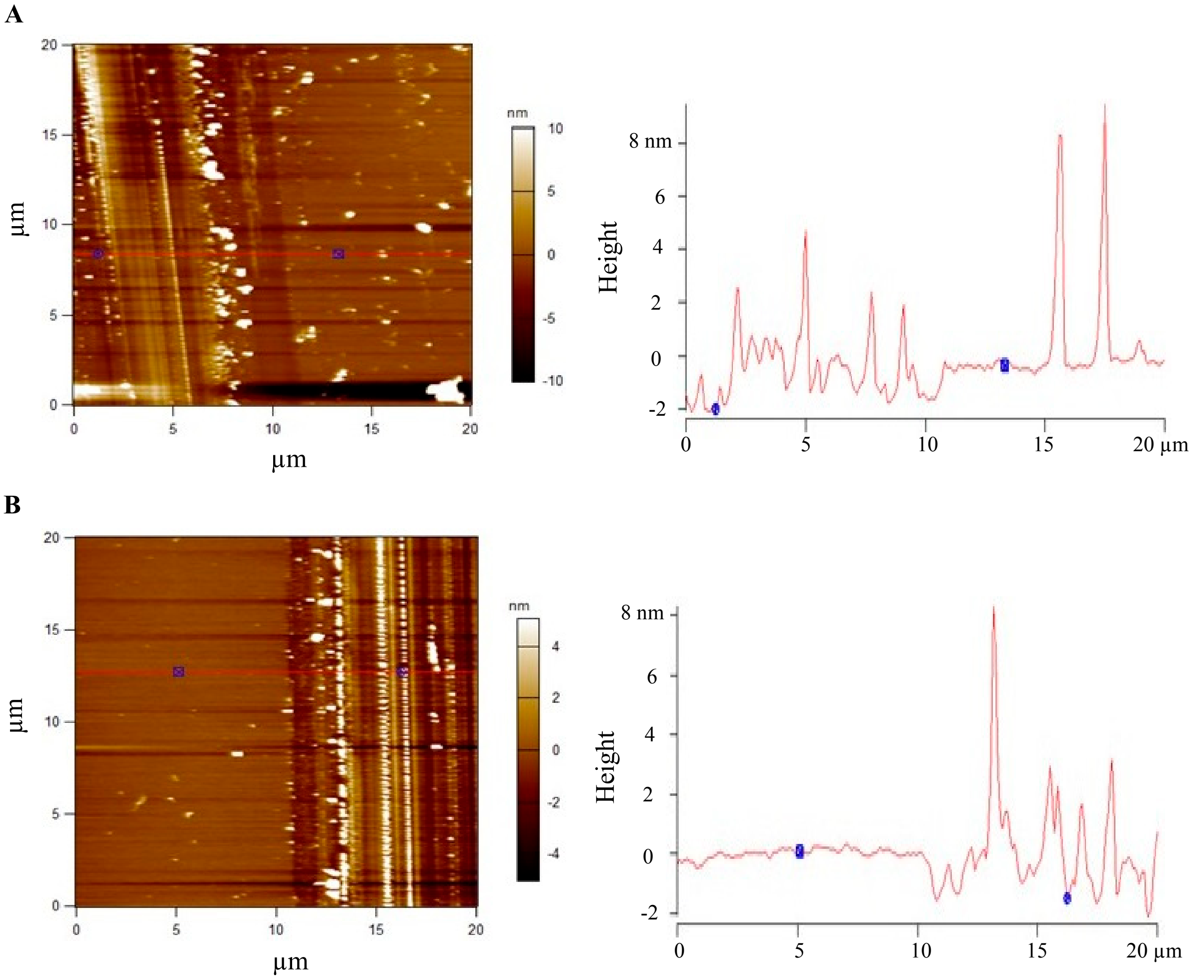

Figure S3. Atomic Force Microscopy (AFM) height (left) and deflection (right) images of the interfacial film formed by Chp F at pH 3.0 (**A**) or pH 10.0 (**B**). The film at the air/water interface was transferred onto a silicon wafer surface prior to imaging. Film thickness was estimated by scratch analysis (mechanical removal of the film) and by tracing a profile along the film and the scratched zone. The interfacial films formed by Chp F at pH 3.0 and 10.0 were found to have a similar thickness of 1.6 ± 0.4 nm, despite the differences in morphology and roughness (676.15 pm vs 136.41 pm respectively). The thickness values represent the mean of at least three measurement areas along the scratch profile with the standard deviation of the three replicate experiments (n = 3 ± standard distribution). These images correspond to a sample area of 20 µm × 20 µm.
